# Mammal’s meat and cow’s milk allergy – case report

**DOI:** 10.1186/2045-7022-3-S3-P152

**Published:** 2013-07-25

**Authors:** F Ribeiro, I Carrapatoso, B Bartolome, A Segorbe Luís

**Affiliations:** 1 Imunoallergology Department, Coimbra University Hospital, Coimbra, Portugal; 2 R&D Department, Bial-Aristegui, Bilbao, Spain

## Background

The mammalian meat allergy is not very common. When it happens in adults, it may be related to respiratory allergy to mammalian epithelia (e.g. pork-cat syndrome) or to sensitization to the recently discovered IgE antibody to a mammalian oligosaccharide epitope galactose-alpha-1,3-galactose (α-gal).

## Methods

We present the case of a 66 year-old woman that at age 61 developed recurrent episodes of generalized urticaria and angioedema of the upper airways about 4 hours after the ingestion of ham, bean stew with pork meat and rabbit meat. She tolerated other foods, including other meats, fish and cow's milk. She worked in a meat factory and handled daily with meat, she had contact with various animals, including dogs, cows, pigs, chickens and doves. Two years after the onset of the clinical manifestations she had other episode after the ingestion of cow’s milk. She avoids all mammalian meat and cow’s milk. No history of respiratory allergy was reported. Skin prick tests (SPT) and determination of specific IgE (sIgE) to aeroallergens, meats and epithelia from mammals, cow’s milk and to α-gal and nBos d 6 were carried out. We performed SDS-PAGE immunoblotting assays to mammalian meats. Open oral challenge to cow’s milk was also performed.

## Results

SPT to aeroallergens were positive to pollen from alder, plantain, mugwort and Parietaria judaica as well as to Alternaria alternata. SPT and sIgE results to allergens other than aeroallergens can be observed in the table. The immunoblotting assays identified an intense IgE-binding band of 60 kDa and some others of high molecular masses. The oral challenge test to cow’s milk was considered positive after a cumulative dose of 200 ml, about 3 hours after last intake.

**Table 1:** 

	sIgE (kU/L)	SPT (mm)
Pork’s meat Cow’s meat Lamb’s meat	3.59 5.36 Nd	5 4 4

Cow’s milk Casein	0.94 0.02	0 5

α-gal nBos d 6	3.17 0.00	Nd Nd

Dog Cat	1.10 <0.35	3 4

## Conclusion

This is a case of a patient with non-immediate anaphylaxis to mammalian meat and cow's milk. The proteins usually involved in type I allergy to mammalian’s meat (serum albumin) and to cow’s milk (casein) were negative. In this case the molecule involved seems to be the oligosaccharide alpha-gal. This may explain allergy reactions to mammalian meats with negative sIgE values to nBos d 6 and allergy to cow’s milk with negative sIgE to milk proteins.

## Disclosure of Interest

F Ribeiro: None declared, I Carrapatoso: None declared, B Bartolome: Employee of R&D Department, Bial-Aristegui, A Segorbe Luís: None declared.

